# Indigenous Narratives of Health: (Re)Placing Folk-Medicine within Irish Health Histories

**DOI:** 10.1007/s10912-014-9322-4

**Published:** 2014-12-09

**Authors:** Ronan Foley

**Affiliations:** Department of Geography, Rhetoric House, National University of Ireland Maynooth, Maynooth, Co. Kildare, Ireland

**Keywords:** Folk medicine, Ireland, Healing, Water cures, CAM

## Abstract

With the increased acceptance of complementary and alternative medicine (CAM) within society, new research reflects deeper folk health histories beyond formal medical spaces. The contested relationships between formal and informal medicine have deep provenance and as scientific medicine began to professionalise in the 19th century, lay health knowledges were simultaneously absorbed and disempowered (Porter 1997). In particular, the ‘medical gaze’ and the responses of informal medicine to this gaze were framed around themes of power, regulation, authenticity and narrative reputation. These responses were emplaced and mobile; enacted within multiple settings by multiple agents and structures over time. The work is drawn from secondary material from Ireland, which identify more indigenous narratives of health and act as potential sources for medical humanities. While assumptions have been made as to the place of folk-medicine being essentially rural, evidence will be presented which shows a more complex network of health beliefs and practices. The narratives of informal practice and folk-medicine drawn from evidence from Ireland point to more fluid and hybrid relations with formal medicine and suggest that the complementary nature of the two models reflected wider cultural debates and models of belief (Del Casino Jnr., Health & Place 10:59-73, 2004).

## Introduction: Informal health histories

In the library at Bailieborough, County Cavan, Ireland, there is a small ring-bound folder in the local studies section which contains a list of folk healers with a specific listing of which ‘cures’ they can perform. It lists a number of informal local healers but also some from a considerable distance away with either a phone number or a brief address. The specific conditions (45 in total) for which the ‘cure’ can be accessed range from arthritis to warts and includes illnesses such as Bell’s palsy, skin cancer and tuberculosis. Entries note that a lady with the cure for hiatus hernia for example, lives in the townland of Crookswood and to find her:

‘Go on to filling station, take left turn, and then left again past school, into narrow lane, half mile, lane on right. Through gate house in wood (niece lives at end of lane). XX is an elderly person. Generally works Sat-Mon-Thurs’ (Bailieborough Library Folder, undated).

The survival of such a listing, well-thumbed I might add, is testimony to a deep connection in Ireland with the concept of the folk cure, something which resides in individual practitioners and in specific places; that is publicly known but privately practiced. While the clinical qualifications and ability of those with the ‘cure’ may not withstand deeper biomedical scrutiny, what matters more is the sense of a long narrative connection between folk medicine, health cure and their co-presence within what health geographers refer to as therapeutic landscapes (Wilson 2003; Williams 2007). This relationship among folk medicine narratives, curative practices and place form the basis of its potential interest for medical humanities research and this paper will discuss these in relation to historical health practices on the island of Ireland (Foley 2010).

Drawing from earlier research on ‘healing waters’, the different practices, practitioners and settings of hydrotherapy are extended in this paper to consider folk medicine more widely (Foley 2010). A brief review of the literature considers where folk medicine sits within therapeutic landscapes research. The strong qualitative and reputational components of therapeutic landscapes research are then discussed. The particular use of narrative sources and imaginative accounts which underpin this research also provide an explicit link to medical humanities. As a final literature component, histories of medicine are discussed in terms of how they are often framed by the relative positionalities of formal and informal medicine though other dualistic terms such as orthodox/unorthodox or conventional/unconventional were equally applied (Price 1981; Porter 1997). Quackery and the potentially harmful practices of the unqualified, unscrupulous and profiteering quack are central themes in these literatures (Porter 1989).

In considering a set of themes in depth, particular concerns emerged around the linked themes of power/regulation and authenticity of both practices and practitioners within Ireland from the 18th to 20th centuries. These were never as black and white in reality and remain contested to the present day. A third theme explicitly links medical/health geography and medical humanities in looking at the reputational narratives of folk medicine. These were explicitly related to practices in place and in turn informed the reputations of places, practices and practitioners in driving and sustaining both local and national economies of folk medicine, still visible today in the thriving markets for complementary and alternative medicine (CAM) (Moore and McClean 2010). A mix of secondary and primary Irish empirical material is used to illustrate these themes, drawn from a range of therapeutic landscapes and practices. Finally, the paper takes a position that sees the medical humanities as a challenge to medicine ‘to become interdisciplinary, and be disciplined by arts and humanities as well as science’ (Bolton 2008, 132). In looking at a range of narratives and themes that sees the practices of folk medicine as having an interdisciplinary intent, the ongoing braiding of multiple strands of medical practice remains a central tenet.

## Literature review

In recent therapeutic landscapes literature, both historic and contemporary discussions on spiritual health, nature cures and general indigenous medical practices have been prominent themes (Wilson 2003; Williams 2007). Therapeutic landscapes are broadly described as places that have achieved lasting reputations for providing physical, mental and spiritual healing and typically include settings such as spa towns, pilgrimage sites and wilderness as well as smaller scale baths and retreat settings (Gesler 2003). Andrews and Kearns’ (2005) layered examination of health histories in place in their study of Teignmouth in the UK, though focused on more formal services, provides a useful template. Providing a potentially valuable parallel link between developments in cultural geography and a wider medical humanities, research by Foley (2011) considers a ‘deep mapping’ of place and the associated practices and inhabitations that form the basis of embedded healing assemblages (Bailey and Biggs 2012). In considering those more profound histories, it may be valuable to recast folk-medicine as representative of a set of traditional public health practices to be set alongside the necessary development of formal health care services (Buckley 1980). Central to this were ‘informal practitioners and practices’ placed somewhere between professional and lay health/medical knowledges. In addition, there were long histories in a number of Celtic countries around the complex relationships between charms, nature cures and the power of hereditary healing families, members of which had a control over the practice of medicine in locations like Ireland and the Highlands of Scotland (Donoho 2012).

Medical historians like Porter (1997) and Kelly (2009) note the writing on informal medical practices as often contradictory, simultaneously appalled and fascinated. In a contemporary setting, similar themes continue to shape work on complementary and alternative health. Examples include the work of Heller et al. (2005) on contested definitions and professional power divisions between CAM and conventional biomedicine. In addition, medical/health geographers have studied contemporary examples of CAM practice around yoga and wellness practices (Hoyez 2007; Lea 2008), in which the spatial networks and narrated meanings of CAM are considered in terms of the reproduction of a set of globalised therapeutic settings/practices. Many of these contemporary CAM themes can be specifically applied to folk medicine, yet there appears to be a reluctance to connect them together explicitly in the literature. It is also important to note the contested nature of such forms of practice especially by formal medicine and a positioning of all forms of non-allopathic medicine as not so much CAM as SCAM (in its literal sense). This too reflects historical work on quackery and in particular the present role of the Internet as a source of ‘inauthentic’ medical knowledge (Doel and Segrott 2003). Yet a counter narrative in the literature in Ireland is evident in work by Moore and McClean (2010) and Patrick Logan (1981), a medical doctor with a deep affinity for folk-medicine and country cures and a coherent advocate of the expertise of its prominent practitioners.

Finally in the medical humanities literature, David Hufford’s (2003) work on belief, spirituality and health also makes for provocative reading. His arguments focus on the invocation of rationality by the scientific medical community as a means to discredit any form of ‘unapproved’ medical practice. Yet, he argues, the act of discrediting is itself an irrational act in that science cannot effectively predict human behaviours, responses and choices. In addition he argues that there are a range of parallel narratives defined by patient beliefs, experimental practices and local traditions of bodily care to be found in all multi-cultural societies (Hufford 2003). In this collective research on CAM and folk medicine, one can identify a number of overlapping themes founded on power/regulation, authenticity and reputational narrative, and these will act as points of discussion across the remainder of the paper.

## Sources

Drawing from a hybrid collection of primarily secondary qualitative sources narrative, reputation and inhabited practice as much as medical evidence play a key part in methodological approaches appropriate to medical humanities research. Sources including travellers’ accounts, archival records, national collections and oral histories are all utilised to identify discourses around folk medicine. In addition, some contemporary ethnographic fieldwork including site visits and participant observation in extant practices, such as at holy wells, were also undertaken. In considering representative material on folk medicine in Ireland, the role of narrative is central. This narrative is reflective of a pragmatic and applied set of healing texts and practices which provide a parallel evidence-base to that used in scientific medicine. That evidence is not only drawn from sources which discuss the position of folk-medicine in relation to its own practices and settings but also frames that discussion against scientific medical structures. A key source in Ireland is the National Folklore Commission’s surveys of 1934–8 which included a particular interest in the term *leighis* (cure), and how these were recorded within specific locations (National Folklore Collection 1934). These collective narratives act as a timely recording of indigenous health knowledges and practices, and the types of cures identified included listings of herbal folk cures, charms and spells as well as accounts of individual healers and their expertise. Seventh sons of seventh sons, for example, were regularly mentioned in archival material as were hereditary skills in bone-setting or the cure of warts, handed down, usually though not always, from father to son (NFC 1938). Health outcomes were recorded for both humans and animals, and these repeated stories drawn from multiple locations ranged from the para-medical to the magical. A second parallel survey undertaken by the Ulster Folk Museum in the 1960s focused specifically on folk-medicine in Northern Ireland. A common historical medical humanities source, external travellers’ accounts, described practices and cures from the perspectives of a range of correspondents, some positive, some hostile (De Latocnaye 1984; Hardy 1836). Often ‘shamrockist’ in their gaze, the travellers were objective and occasionally bewildered recorders of the practices they observed.

Other essentially oral sources, especially relevant given that many cures were enacted through speech, included old songs, poems and charms passed down by *seanchaí*, recognised local storytellers. Examples included interviews from 1979–80 with older residents about folk cures in the Dublin and Wicklow Areas (NFC 1980). Finally, material from local historical journals, often overlooked in academic research, provide much of the depth in a deep mapping of place-based health practices. In terms of local narratives of folk medical practice, these accounts via oral testimonial sources are valuable in themselves but also record contestation from more official forms of medical practice. Overall, the sources for folk medicine are often partial and ephemeral in contrast to the records of a more professionalized and centralized medical profession. This was in part shaped by the location (often, but not exclusively rural) and nature of the practice of folk-medicine as a collective and at times lay practice, in which familial knowledge, oral traditions and a communal attachment all played a part (Bourke 2001).

## Power and regulation

When considering folk medicine in Ireland across the 18th to 20th centuries, power was a central theme, especially in terms of its position within the wider practice of formal medicine and healing. Linked to power, regulation played a significant role, in different forms, in the management of that power. Foucault noted that there was a quite blurred history within what he termed, noso-politics, in how formal medical structures took hold and older folk practices were subsumed or incorporated to a wider public health from the 18th century on (Foucault 1980). Hierarchies were evident in the expression of power between both informal and formal practitioners in place and also around patient/practitioner interactions. From the 18th to well into the 20th century, there was a contrast between local, often free healers, and the slowly developing professional for-profit medicine. In rural Ulster, the hierarchies were subverted somewhat in rural areas because of a preference for local folk healers and a deep distrust of the ‘collar and tie men’ of the medical profession (Buckley 1980). At the other end of the scale, the professional bodies responsible for scientific medicine created an identifiable group of trained professionals for whom the practices of folk medicine seemed anathema. Yet such a positionality of inclusion and exclusion was never clear-cut in terms of the experience of health care provision and utilization across the country, evident in the different spaces of practice and the overlapping bodies of practitioners.

**Fig. 1:**
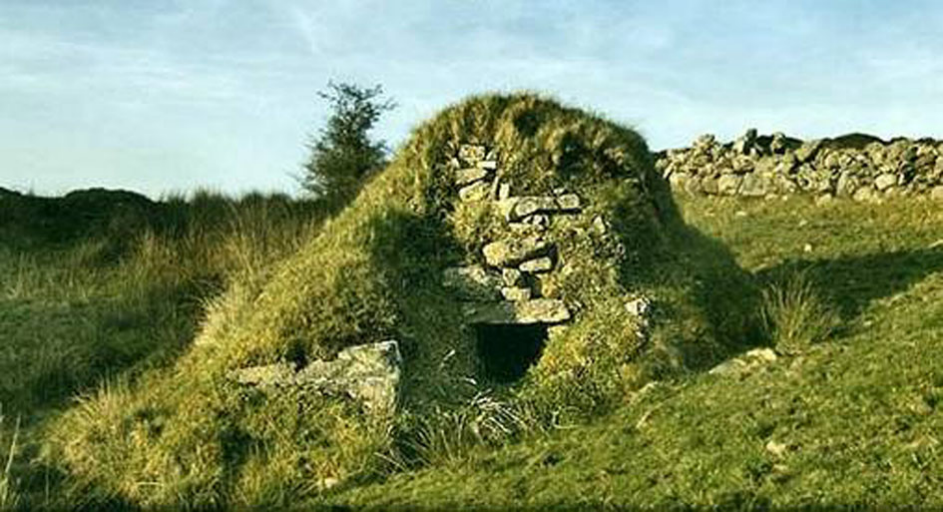
Sweat House. Legeelan, County Cavan (Source: Author)

In considering the relationship between power and sites of medical practice, it was expressed in a geography that was hierarchical and relatively rigid. Spatially, power needs to be concentrated to be visible, hence the symbolic importance of the hospital or workhouse in Irish research. Yet the more fluid practices of folk-medicine were expressed in mobile sites and settings, certainly in terms of some of the belief-based practices; conditional, relational, even sometimes invisible in that knowledge of their existence or location only existed via word-of-mouth. Such settings included country fairs, people’s homes, the healers’ own homes as well as other communal settings, but all were places associated with a reputational form of healing power and energy. Empirically the sweat house was a good local example (Evans 1957). As small constructions dotted across the landscape, sweat houses were an essential folk-medical site in rural upland areas (Foley 2010). Looking a little like stone igloos covered by grass and earth, the interiors were heated with turf, and patients entered and spent time in the closed settings and sweated out their fevers (Fig. 1). An account from Rathlin Island on the north coast of Antrim noted:

‘…that previous to the bath, a fire was kindled inside, and when it was sufficiently heated, the ashes were swept out. The people came to be cured of the *pianta fuar*, as she called the rheumatism, the Irish name meaning literally ‘cold pains.” (Mulcahy 1903, 589).

While one of the concerns of formal medicine was the lack of regulation they associated with folk medicine, there was evidence of some good regulatory practice at sweat houses (Hufford 1998). Used to cure flu, arthritis and rheumatism, they were sometimes regulated by itinerant bath masters who would check potential users as to their ability to withstand the rigours of the sweating cure (Richardson 1939). More importantly, sweat houses were privately or communally owned, providing a service to extended families and small communities in remote locations especially in the northern half of the country (Foley 2010). This was especially important in locations where any form of conventional primary health care service did not meaningfully emerge until the end of the 19th century when a network of dispensaries, (noted by Foucault (1980) as part of a new ‘medico-administrative’ apparatus of power), introduced a more regulated set of public health spaces into the Irish countryside. But up to this time, sweat houses, similar in form to Scandinavian sauna or Mexican *temazcalli*, developed epigenetically and provided a form of local empowerment and ownership over a set of necessary healing practices (Groark 2005).

**Fig. 2:**
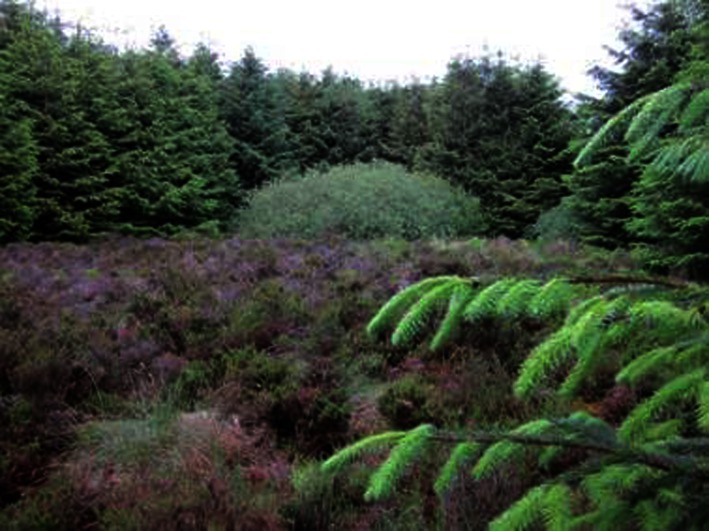
Site of Lough Leighis (Loughanleagh), County Cavan (Source: Author)

As a second example, there were a range of sites associated with water which formed an important component of the folk-medical geographies of Ireland. Holy wells were one classic form, a mix of spiritual and physical healing site mentioned in the introduction and discussed in more depth elsewhere (Foley 2011). Another example, Lough Leighis, was a famous healing lake in East Cavan, visited up to the 20th century by users who came from long distances to take away its curiously curative and energizing mud. It is now buried under bog and forest (Fig. 2) but encapsulates a setting associated with a perceived natural curative substance, akin to herbal medicines found in most cultures*.* The mud from the lake had a particular reputation for curing skin diseases including scurvy and leprosy and indeed was distributed around the country as a curative product (Coote 1802; Kelly 2009). This local nature-based collection of therapeutic materials echoes what Kathi Wilson refers to as the ‘24-hour pharmacy’ in relation to the Canadian First Nations term for the land as a source of curative berries and herbs (Wilson 2003).

**Fig. 3:**
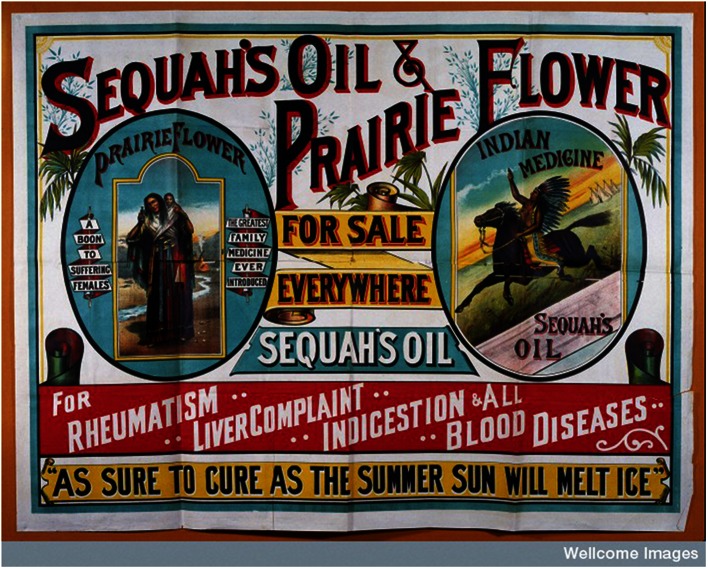
Sequah Poster (Source: Wellcome Images)

A persistent associated theme in discussions of medical regulation and power was that of training and healing expertise. In the development of a hierarchical structure of formal medical power, training was crucial. Metaphors exist of the folk practitioner as representing the ignorant/untrained/low/unapproved and the medic as representing the knowledgeable/trained/high/approved (Logan 1981; Moore and McClean 2010). Such words recur over and over in discussions, evidence of a notional ‘drawbridging’ of ownership and status. The relationship between training and regulation can even be seen in contemporary attempts by CAM practitioners to be accepted by formal medicine through strict training requirements (Clarke et al. 2004). Critical questions do need to be asked about the regulation of informal folk medicine as accusations of quackery were often apposite (Porter 1989). Quackery did result in some genuinely dangerous historic practices, the response to which in turn became a central plank in medical regulation and the creation of professional bodies. There were some very dubious practitioners in the towns and countrysides of the British Isles from the mid 19th century on. One example, a form of hereditary quackery franchise, was to be seen in the presence of travelling healers called Sequahs (Schupbach 1985). These Sequahs--there were twenty-seven of them over time and they operated across the British Isles--built an identity for their practices and products based on American Indian healing and commodified through their exotic medicines, which though folk were not exactly local (Schupbach 1985). Most were English born, unqualified and sold a patent medicine, Prairie Flower oil, which was proven to have no medical benefit at all (Fig. 3). While their business was eventually declared illegal, they still attracted audiences despite a patent lack of training or medical regulation. What was perhaps most interesting about the sequahs was their exotic nature, which while ‘folk’ was certainly neither local nor indigenous. Yet at the same time as they practiced their trade in Irish towns such as Dublin and Kilkenny, there were other rural practitioners trading in patented rubs and potions who were less visible, but importantly less commercially motivated (Maloney 1972; Fleetwood 1990).

## Authenticity

Despite strong relationships between regulation and professional training, authenticity and ownership of practice have always been slippery themes in medical history. In theoretical terms, debates on meta-narratives lie at the heart of cultural health geographies and emphasise the need to consider a more heterogeneous story. Hufford (1998) for example, suggests the need to consider a ‘methodological populism’ (302), which assumes an equal value for all forms of health narrative and practice. Here the practices of formal and informal medicine were arguably much more connected than they might seem. There was an overlapping use of knowledge bases, and trite though it might seem in any notional performance of health, there was a sense that ‘practice made perfect’. In folk medicine terms, this meant that many traditional healers had access to and used existing medical texts, photocopied, passed on through generations of families with as Logan (1980) observes, extensive liner notes. The practice makes perfect notion was as much a feature of the work of the traditional bone-setter as the contemporary surgical rotation. Indeed as an empirical example of authentic practice, the bone-setter was and remains an important folk practitioner across cultures and has strong links to contemporary authenticated forms of CAM like osteopathy and chiropractic (Heller et al. 2005). In Ireland, the bone-setter was a valued folk medical practitioner across the province of Ulster. While they often carried out an itinerant practice, in that they moved from place to place as they were needed, they also operated from known locations to which they drew in turn an itinerant clientele (Buckley 1980). Almost always male, they drew on a wealth of often hereditary experience, learnt from their fathers and grand-fathers which as far as their patients were concerned, gave them as much authenticity and ownership of practice as allowed them to continue to heal.

A second feature of the authenticity of folk medicine practices, associated also with contemporary biomedical practice in secondary care settings, was the enhancement of practitioner expertise to ensure what one might term ‘clinical mass’. Logan (1981) describes the unacknowledged expertise of the informal practitioner though an extensive case-load and genuine physician training as being in itself an authenticating practice. Here the experientially-developed knowledge underpinned a subsequent therapeutic reputation, which in turn was drawn from that repeated practice. Logan, himself a medical doctor, also commented on the regard of formal doctors for the informal doctor’s skills and suggested within his own country practice that even in the 1980s a form of cross-referral was taking place. Indeed a bone-setter in County Carlow with forty years experience was invited to speak to physicians in formal training sessions in the early 2000s while similar evidence of referral from general practitioners to informal healers was found in Mid-Ulster in 1985/6 (Naughton 2004; Moore 2010).

**Fig. 4:**
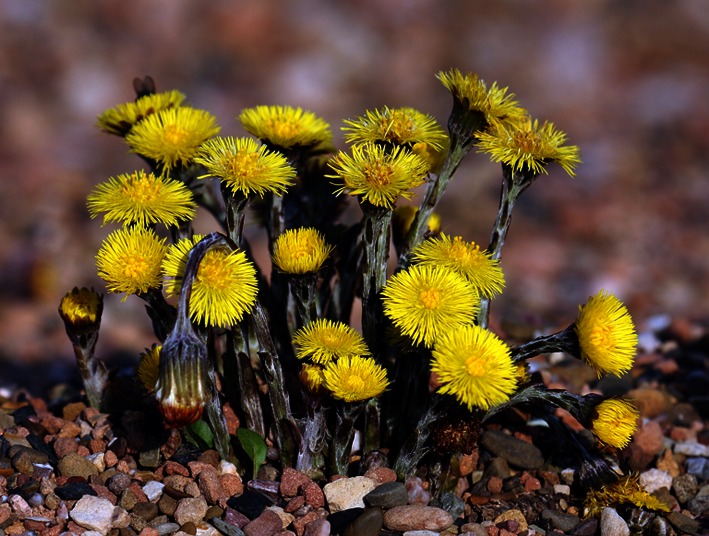
Coltsfoot (Source: Andreas Trepte, www.photo-natur.de)

In considering a demand for folk medicine, Cox’s (2010) description of the medical marketplace in Ireland from 1750 to 1950 demonstrated the existence of a hybrid setting where folk and scientific cures were equally used, a forerunner perhaps of the online medical marketplaces of the early 21st century. Herbal medicines were central to folk-medicine and drew from pharmacopeias of considerable range. The violet was used in Ulster as a cure for cancer by dint of stewing and drinking of the liquid and the same applied to coltsfoot (Fig. 4) as a cure for asthma and lung disorders (Ballard 2008). This simple form of extracting concentrated therapeutic materials from original herbal form would not be unfamiliar to a modern pharmacist. Poultices, evident in forms such as eel-skin bandages from Lough Neagh, have parallels in many cultures (Ballard 2008). In such rural settings, the relationships between the authenticity of a folk medical practice/cure was cemented by their perceived efficacy as well as a place-specific sourcing of materials considered to be curative.

Extending that utilisation perspective, the question also emerged as to who authenticated the practice--the medic or the patient? This applied particularly to gendered bodies of knowledge and in particular narratives of birthing and midwifery practices, engaged in by a range of unregulated but far from ‘unexpert’ midwives and local informal birthers. In addition, the waters of holy wells and their associated rituals were used in a range of reproductive practices ranging from assistance in conception to delivery and post-natal care (Foley 2010). Two wells visited by the author in 2008 and 2009 showed how these narratives sustain into the present day. St. Patrick’s Well in Clonmel--shaped like a womb--was and is visited by couples planning to have children as recounted by a local guardian, while Fr. Moore’s Well in Kildare has long been used for post-natal blessings, particularly among members of Ireland’s Travelling Community, a practice observed during those visits. This gendered perspective, especially as it relates to an embodied knowledge is certainly one which persists in a range of indigenous settings, where local cultural traditions associated with women’s health have long and authentic histories (Cross and MacGregor 2010). Hufford also notes this as an especially good example of ‘inappropriate notions about the boundaries of expert knowledge and authority’, contrasting biomedical constructions of childbirth as a medical emergency against a reassertion by women of the authority of experience and traditional knowledge (1998, 302).

## Reputational narratives

**Fig. 5:**
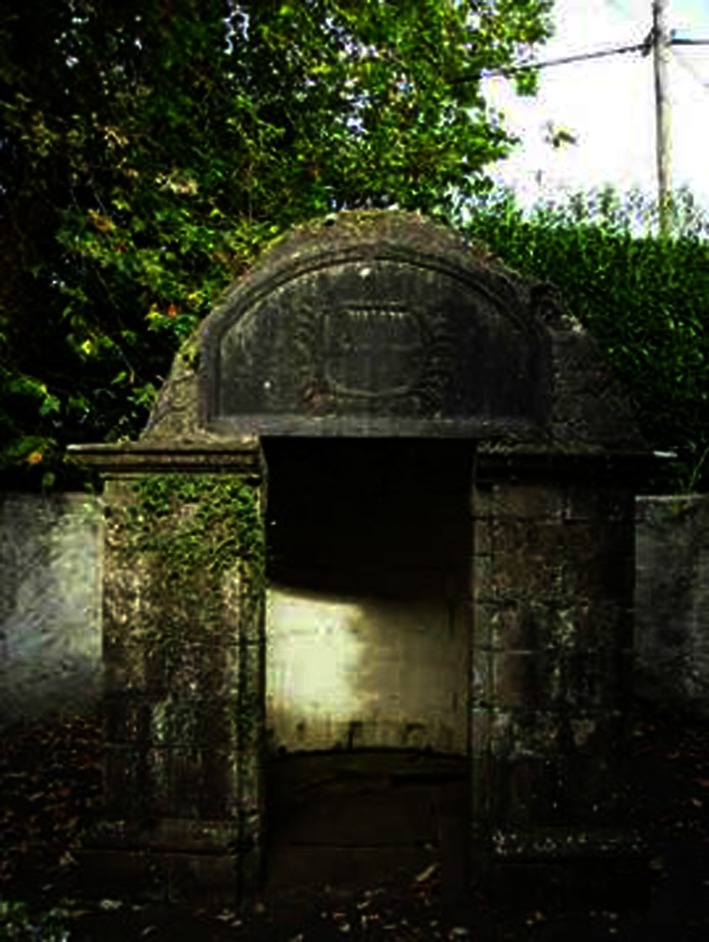
Covered Water Source, Castleconnell Spa, Co. Limerick. (Source: Author)

In considering a wider medical humanities, reputational narratives were central to the perceived value of folk medicine, especially as it was to be found in therapeutic landscapes. Just as the therapeutic landscape of the spa or holy well relied on its sustained healing reputation, so the reputation of the folk practitioner also depended on word-of-mouth and indeed, stories of effective cures associated with that practice. If a neighbour or friend came back cured or in less pain for a particular condition, that was physical evidence of an effective practitioner. Ineffective or harmful practitioners might have their performance perceived as ‘losing their touch’, as a loss of healing energy. One might suggest that this concern with consistently measurable health outcomes has some interesting parallels with contemporary hospital league tables. In empirical terms, a whole history of spa medicine, considered scientific in its time but subsequently relegated to the margins of practice, was visibly focused on the reputations and narratives of the water. The quality of the water and the quality of the cure were an essential aspect of their popularity, and a material spectral trace of the water source at Castleconnell in County Limerick, considered effective for liver complaints, jaundice and ulcers, is pictured in Fig. 5 (Rutty, James 1757). This hybrid space, where informal and then-formal medical practices overlapped, was one in which both orthodox and non-orthodox narratives took root.

**Fig. 6:**
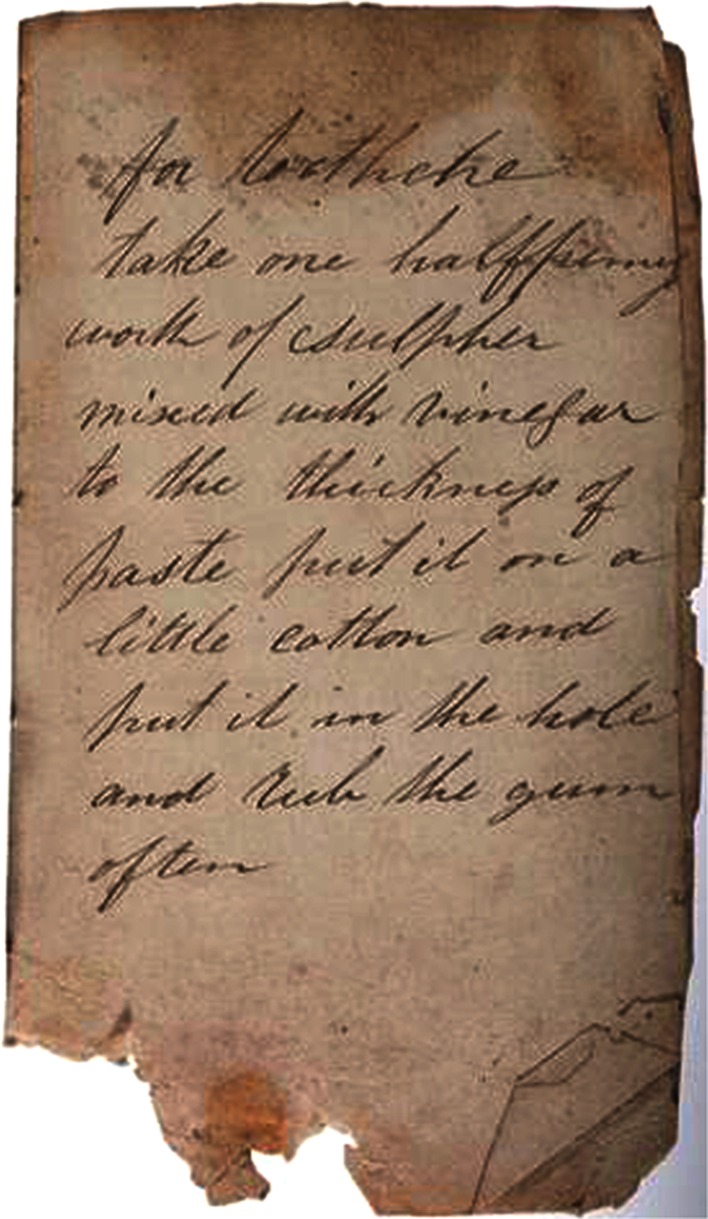
Toohtache Cure written on the back of an envelope (Source: NFC)

The narrative as text was also embedded directly into practice. The idea of an ‘oral pharmacon’, words that could both kill or cure, was an important concept in Ireland. Many of the cures, especially in Ulster, were expressed as ‘charms’, healing words to soothe the patient and invoke a form of external healing potential (Ballard 2008). Old Irish texts used a term also used in mainland Europe, the ‘*éle*’, to represent an oral cure that could be curative and also maledictive. The texts were used in combination with the medicine and the practitioner. Curative texts can also be found in the various folklore collections, and one example is a written cure from the 1870s for toothache, written on the back of an envelope in Longford (Fig. 6).

This relationship between the written and spoken word was an important component within Irish folk medicine narratives. In the 18th and through much of the 19th century, the Irish population were not especially literate, so an oral culture was central to the recording and communication of folk medical knowledges (Buckley 1980). The ‘cure’ was often an incantation or spoken treatment, echoing the idea that a ‘talking cure’ in Ireland was part of a wider global culture of indigenous healing power wherein an oral medicine was managed by a wide range of shamans, priests, healers and wise elders. Moore (2010) recounts an ongoing practice in Mid-Ulster from the mid 1980s of cures or charms as they were referred to locally, which he identified as in part oral but which operated with the tacit approval of local general practitioners and which formed part of a wider social transaction.

While assumptions have been made as to the place of folk-medicine being essentially rural as opposed to the rational and advanced city, evidence shows a more complex network of health beliefs and practices. Many examples of folk cures in the NFC came from Cork, Galway and Dublin with detailed written descriptions for how visitors in search of a cure should behave at a holy well. In many cases this consisted of a list of prayers, walking circuits and physical actions which were a necessary part of a cure negotiation in place (Foley 2010). To receive the full benefits, one had to obey the rules, whereas a transgression would result in no cure or even a worsening of one’s condition. People who used holy wells incorrectly or who interfered with well tradition generally got a shock or unpleasant surprise. At St. Kieran’s Well in County Meath, the entry in the NFC Collection noted the effect of an interference with the legends associated with the well:

The pattern day of St. Kieran is the first Sunday of August. At midnight of the previous night there is usually a crowd around the well waiting for the three trouts which are supposed to appear at 12 o clock. People say they are hundreds of years old. Once a man happened to catch them, brought them home and put them in the pan. They hopped off and were next seen in the well. (NFC 1934)

In 2012, in conjunction with a colleague from Trinity College Dublin, a testing programme of three historic spas and twelve holy wells across Ireland was carried out. The programme (part of a postgraduate research project in hydrogeology) was partially an investigation of chemical concentrations in water; it was equally motivated by testing whether folk narratives of specific cures at holy wells and spas had any basis in scientific medicine. The results were mixed, as much from the lack of local cure narratives as the presence of high levels of curative minerals. However, a number of interesting results emerged, especially at a famous holy well in County Kerry, *Tober na nGealt* (located in *Gleann na nGealt*, the Valley of the Insane). The chemical testing identified forty times the normal concentrations of lithium, an established contemporary chemical used in the treatment of mental ill-health. In aligning this with the folk medical narratives, people had been coming (and often left here) for centuries, as both well and valley had established reputations for curing madness (Logan 1980).

The survival of folk medical practices were also arguably linked to migration, a form of ‘cura in urbis’, where rural traditions and practices were relocated and survived within urban settings. This too can be extended to contemporary work on CAM practice amongst migrant communities (Cross and MacGregor 2010). This final thematic concern for narrative-based medicine brings us back full circle to the contested relationships between scientific and talking cures and the increased attention and acceptance within medical humanities of narrative and belief-based medicine. Indeed in the discussions on folk medicine in Ireland, classic imaginative texts such as Carleton’s Gothic novel, *The Evil Eye*, provide a rare account of behaviours at an Irish Spa town, Ballyspellan.

## Discussion and summary

In thinking about deep mappings of healing, folk medicine geographies emerge as mobile, relational, complex and persistent. The narratives of informal practice and folk-medicine drawn from Irish evidence suggest fluid and hybrid relations with formal medicine, and the complementary nature of the two models reflects wider cultural models of curative belief that are important to the wider field of medical humanities. Del Casino Jr. (2004), commenting on indigenous health in Thailand, notes that health acts as a marker of contested social relations enacted through place and sees it as a struggle between local knowledge and global change. In Ireland, this was evidenced by the decline of holy well and sweat house use for example, by the introduction of a network of dispensaries and country doctors in the later nineteenth century. Contemporary globalising therapeutic landscapes and practices such as yoga, ayurveda and spa retreat spaces seem to favour the exotic, an arguably ‘Orientalist’ CAM that differs a little from a more local and genuinely complementary CAH (complementary and alternative health). In addition, folk medicine has always been seasonal/temporal/relational/local with regard to its curative potential. In utilizing Wilson’s (2003) notion of the 24-hour pharmacy, it might be suggested that in nature the folk medical pharmacy may never close but its shelves are stocked differently depending on the season.

The position of folk medicine, both as form and practice, suggest the co-presence of a set of emplaced and embodied energies of health. Many of the cures were place and object specific, from which energy was drawn and reoriented towards the place and the body in the place. For example, many of the rural cures at sweat houses and others sites aimed to provide new energies for work. Reinvigoration was a common narrative in spa settings and was employed in producing in such places the revitalized and re-energised body, visibly getting the flow going within vascular and reproductive systems. In any discussion of health the notion of an ‘oscillation of health’ would be a component part (Bergdolt 2008; Foley 2010). These flowing energies were embodied, yet traditional healers were well-placed in their peripatetic wanderings to tap in to those oscillating health energies. There was also a sense of practitioner energies ebbing and flowing through their practice but simultaneously the sources of folk-medicine doing the same across the year. There were seasonal cures for seasonal illnesses, suggesting a sense of renewable, even sustainable medical energies and products. This contrasts with contemporary high-tech medicine and its considerable energy demands in terms of technology/expertise/speed/power, the direct opposite of folk-medicine as a form of ‘Slow Health’.

There are good reasons why folk-medicine functioned in both historic and contemporary societies, linked to core concepts of accessibility and service gaps, and what emerged organically to fill natural and socially constructed gaps. It was also instructive to note that many Irish practitioners who had the ‘cure’ did not charge for services or left it open for the patient to offer a token payment, a choice not available at the paid general practitioner’s surgery. Again contrasts may be made with the highly commodified contemporary CAM marketplace, but as Moore (2010) noted, there was also a sense of cultural exchange and social support in a set of healing practices that were public beliefs yet private acts. Finally, in considering resilience and resistance, folk medicine in Ireland and elsewhere is partially framed by an instinctive resistance to the ‘conduct of conducts’ of individual health. The slightly dubious contemporary democratization of personal health knowledge via the internet is not dissimilar in nature – if entirely different in form – to the traditional folk healer who also drew on then extant medical knowledges to build up his or her own expertise and user communities. In considering how folk medicine has sustained in the face of a range of hostile gazes, to be displaced is not necessarily to be erased, and resilience remains a feature of both the human body and the social practices of healing and well-being.

## References

[CR1] Andrews G, Kearns R (2005). Everyday Health Histories and the Making of Place: The Case of an English Coastal Town. Health & Place.

[CR2] Bourke, Angela. 2001. “Introduction.” In *FishStoneWater. Holy Wells of Ireland*, edited by Angela Rackard, and Liam O’Callaghan, 7–12. Cork: Atrium.

[CR3] Bailey J, Biggs I (2012). Either Side of Delphy Bridge: A deep Mapping Project Evoking and Engaging the Lives of older Adults in Rural North Cornwall. Journal of Rural Studies.

[CR4] Bergdolt, Klaus. 2008. *Wellbeing. A Cultural History of Healthy Living*. Cambridge: Polity.

[CR5] Bolton G (2008). Boundaries of Humanities: Writing Medical Humanities. Arts and Humanities in Higher Education.

[CR6] Buckley AD (1980). Unofficial Healing in Ulster. Ulster Folklife.

[CR7] Carleton W (1860). The Evil Eye or The Black Spectre.

[CR8] Clarke D, Doel M, Segrott J (2004). No alternative? The Regulation and Professionalization of Complementary and Alternative Medicine in the United Kingdom. Health & Place.

[CR9] Coote C (1802). Statistical Survey of the County of Cavan.

[CR10] Cox, Catherine. 2011. “The Medical Marketplace and Medical Tradition in Nineteenth Century Ireland.” In *Folk Healing and Health Care Practices In Britain and Ireland: Stethoscopes, Wands and Crystals*, edited by Ronnie Moore and Stuart McClean, 55–79. Oxford: Berghahn.

[CR11] Cross, Jamie. and Hayley MacGregor. 2010. “Knowledge, Legitimacy and Economic Practice in Informal Markets for Medicine: A Critical Review of Research.” *Social Science & Medicine* 71:1593–1600. 10.1016/j.socscimed.2010.07.040 20855143

[CR12] De Latocnaye C (1984). A Frenchman’s Walk through Ireland 1796–7.

[CR13] Del Casino Jnr., Vincent. 2004. “Replacing Health and Health Care: Mapping the Competing Discourses and Practices of ‘traditional’ and ‘modern’ Thai Medicine.” *Health & Place* 10:59–73. 10.1016/s1353-8292(03)00019-4 14637287

[CR14] Doel M, Segrott J (2003). Beyond Belief? Consumer Culture, Complementary Medicine, and the Dis-ease of Everyday Life. Environment and Planning D.

[CR15] Donoho, Emily. 2012. “Appeasing the Saint in the Loch and the Physician in the Asylum: The Historical Geography of Insanity in the Scottish Highlands and Islands from the Early Modern to Victorian Eras.” Unpublished PhD thesis: University of Glasgow.

[CR16] Evans E (1957). Irish Folk Ways.

[CR17] Fleetwood J (1990). Irish Quacks and Quackery. Dublin Historical Record.

[CR18] Foley R (2010). Healing Waters: Therapeutic Landscapes in Historic and Contemporary Ireland.

[CR19] ------. 2011. “Performing Health in Place: The Holy Well as a Therapeutic Assemblage.” *Health & Place* 17 (2): 470–479. 10.1016/j.healthplace.2010.11.014 21195654

[CR20] Foucault, Michel. 1980. *Power/Knowledge: Selected Interviews and Other Writings, 1972*–*77.* Edited by Colin Gordon. Hemel Hempstead: Harvester.

[CR21] Gesler W (2003). Healing Places.

[CR22] Groark K (2005). Vital Warmth and Well-being: Steambathing as Household Therapy among the Tzeltal and Tzotzil Maya of Highland Chiapas, Mexico. Social Science & Medicine.

[CR23] Hardy PD (1836). The Holy Wells of Ireland.

[CR24] Heller T, Lee-Treweek G, Katz J, Stone J, Spurr S (2005). Perspectives on Complementary and Alternative Medicine.

[CR25] Hoyez A-C (2007). The ‘World of Yoga’: The Production and Reproduction of Therapeutic Landscapes. Social Science and Medicine.

[CR26] Hufford D (1998). Folklore Studies Applied to Health. Journal of Folklore Research.

[CR27] ------. 2003. “Evaluating Complementary and Alternative Medicine: The Limits of Science and of Scientists.” *Journal of Law, Medicine and Ethics* 31 (2): 198-203. 10.1111/j.1748-720x.2003.tb00081.x 12964264

[CR28] Kelly J (2009). ‘Drinking the Waters’: Balneotherapeutic Medicine in Ireland, 1660–1850. Medicine in 17th and 18th century Ireland’. Studia Hibernica.

[CR29] Lea J (2008). Retreating to Nature: Rethinking ‘Therapeutic Landscapes’. Area.

[CR30] Logan P (1980). The Holy Wells of Ireland.

[CR31] ------. 1981*. Irish Country Cures*. Belfast: Appletree Press.

[CR32] Maloney B (1972). Traditional Herbal Cures in County Cavan, Part 1. Ulster Folklife.

[CR33] Moore, Ronnie. 2010. "A General Practice, A Country Practice: The Cure, the Charm and Informal Healing in Northern Ireland.” In *Folk Healing and Health Care Practices In Britain and Ireland: Stethoscopes, Wands and Crystals*,” edited by Ronnie Moore and Stuart McClean, 104–129. Oxford: Berghahn.

[CR34] Moore R, McClean S (2010). Folk Healing and Health Care Practices In Britain and Ireland: Stethoscopes, Wands and Crystals.

[CR35] Mulcahy, David. B. 1891. “An Ancient Irish Hot-Air Bath, or Sweat-House, on the Island of Rathlin*.” Journal of the Royal Society of Antiquaries of Ireland* XXI (7): 589-590.

[CR36] National Folklore Collection (NFC). 1934. “Manuscript 467.” Cúntaisí ar Thoibreacha Beannuithe do fuairtear ó Mhúinteorí ar fuaid Chúige Laighean. Dublin: National Folklore Collection.

[CR37] National Folklore Collection (NFC). 1980. “Interview with Ann Carroll: Manuscript 199 (Sound Recording).” Urban Folklore Project. Dublin: National Folklore Collection.

[CR38] Naughton, Celine. 2004. “Watch your Back.” *Irish Independent*, September 24.

[CR39] Porter R (1997). The Greatest Benefit to Mankind: A Medical History of Humanity from Antiquity to the Present.

[CR40] ------. 1989. *Health for Sale: Quackery in England 1660–1850*. Manchester: Manchester University Press.

[CR41] Price R (1981). Hydropathy in England, 1840–1870. Medical History.

[CR42] Richardson, Peter. 1939. “Sweathouses between Blacklion and Dowra, County Cavan.” *Ulster Journal of Archaeology* Third Series 2:30–35.

[CR43] Rutty, James. 1757. *An Essay towards a Natural, Experimental and Medicinal History ofthe Mineral Waters of Ireland*. Dublin: No publisher.

[CR44] Schupbach W (1985). Sequah: An English ‘American Medicine’ -Man in 1890. Medical History.

[CR45] Williams A (2007). Therapeutic Landscapes.

[CR46] Wilson K (2003). Therapeutic Landscapes and First Nations peoples: An Exploration of Culture, Health and Place. Health & Place.

